# Supplementary material for: Pf-HaploAtlas: an interactive web app for spatiotemporal analysis of Plasmodium falciparum genes
Source: Bioinformatics. 2024 Nov 20;40(11):btae673. doi: 10.1093/bioinformatics/btae673 (PMC11588202; doi:10.1093/bioinformatics/btae673)

**Supplementary figures**

| **Supplementary Figure 1.** Abacus plots of various haplotypes of interest in the *crt* gene. |
| --- |

#
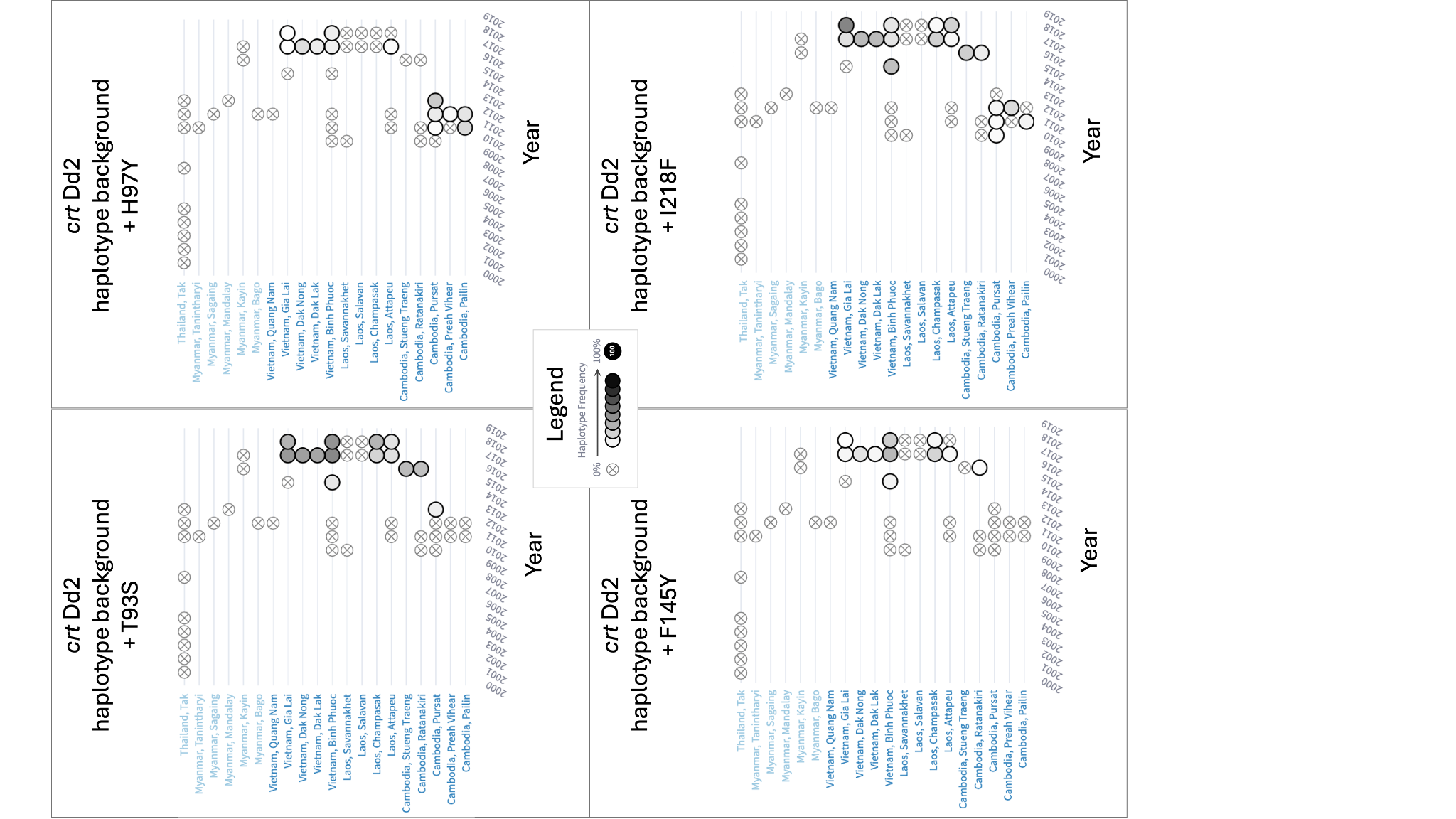

Supplement: btae673_Supplementary_Data [file btae673_supplementary_data.zip › pf-haplotlas 07Nov2024 supplementary.docx]
