# Supplementary figures and images for: Pf-HaploAtlas: an interactive web app for spatiotemporal analysis of Plasmodium falciparum genes
Source: Bioinformatics. 2024 Nov 20;40(11):btae673. doi: 10.1093/bioinformatics/btae673 (PMC11588202; doi:10.1093/bioinformatics/btae673)

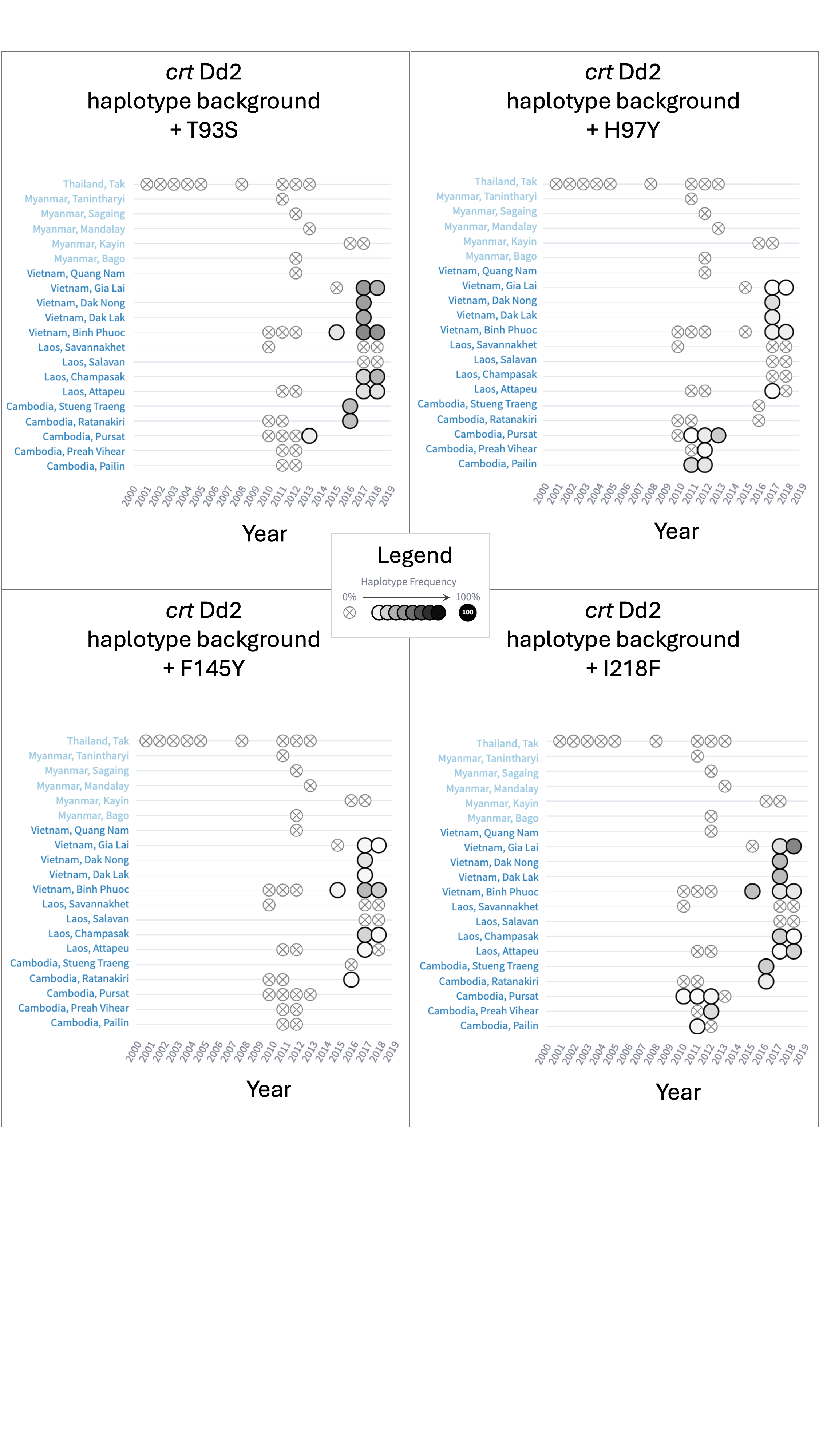

Supplement: btae673_Supplementary_Data [file btae673_supplementary_data.zip › supplementary-figure.png]
